# Long-read transcriptome data for improved gene prediction in *Lentinula edodes*

**DOI:** 10.1016/j.dib.2017.09.052

**Published:** 2017-09-27

**Authors:** Sin-Gi Park, Seung il Yoo, Dong Sung Ryu, Hyunsung Lee, Yong Ju Ahn, Hojin Ryu, Junsu Ko, Chang Pyo Hong

**Affiliations:** a Theragen Etex Bio Institute, Suwon 16229, Republic of Korea; b Department of Biology, Chungbuk National University, Cheongju 28644, Republic of Korea

**Keywords:** RNA-Seq, whole transcriptome sequencing, GFF, general feature format, Gene model, Gene prediction, *Lentinula edodes*, PacBio Single-molecule real-time (SMRT) transcriptome sequencing

## Abstract

*Lentinula edodes* is one of the most popular edible mushrooms in the world and contains useful medicinal components such as lentinan. The whole-genome sequence of *L. edodes* has been determined with the objective of discovering candidate genes associated with agronomic traits, but experimental verification of gene models with correction of gene prediction errors is lacking. To improve the accuracy of gene prediction, we produced 12.6 Gb of long-read transcriptome data of variable lengths using PacBio single-molecule real-time (SMRT) sequencing and generated 36,946 transcript clusters with an average length of 2.2 kb. Evidence-driven gene prediction on the basis of long- and short-read RNA sequencing data was performed; a total of 16,610 protein-coding genes were predicted with error correction. Of the predicted genes, 42.2% were verified to be covered by full-length transcript clusters. The raw reads have been deposited in the NCBI SRA database under accession number PRJNA396788.

**Specifications Table**

**Table:** 

Subject area	Biology
More specific subject area	Genomics and Bioinformatics
Type of data	Table, Figure, GFF
How data was acquired	PacBio single-molecule real-time (SMRT) transcriptome sequencing and evidence-driven gene prediction
Data format	Raw, analyzed
Experimental factors	RNA isolation, cDNA library construction and PacBio sequencing
Experimental features	Long-read transcriptome data with variable lengths were generated, and evidence-driven gene prediction was performed based on the data.
Data source location	The monokaryotic B17 strain of *Lentinula edodes* (KCTC46443) was collected from the Korean Collection for Type Cultures (KCTC) in the Republic of Korea (http://kctc.kribb.re.kr/)
Data accessibility	Raw data from this study are available in NCBI's Sequence Read Archive (SRA) database under accession number PRJNA396788 (https://www.ncbi.nlm.nih.gov/bioproject/PRJNA396788/)

**Value of the data**

• The whole-genome sequence of *L. edodes* has been determined with the objective of discovering candidate genes associated with agronomic traits [1], but experimental verification of gene models with correction of gene prediction errors is lacking.
• PacBio long-read transcriptome data integrated with Illumina short-read RNA-Seq data can enhance the accuracy of gene prediction with error correction and support experimental verification.
• Our data will strengthen genome-wide analyses of *L. edodes* by contributing to the identification of targeted genes associated with a trait, transcriptome profiling, and comparative genomics.

## 1 Data

**Fig. 1:**
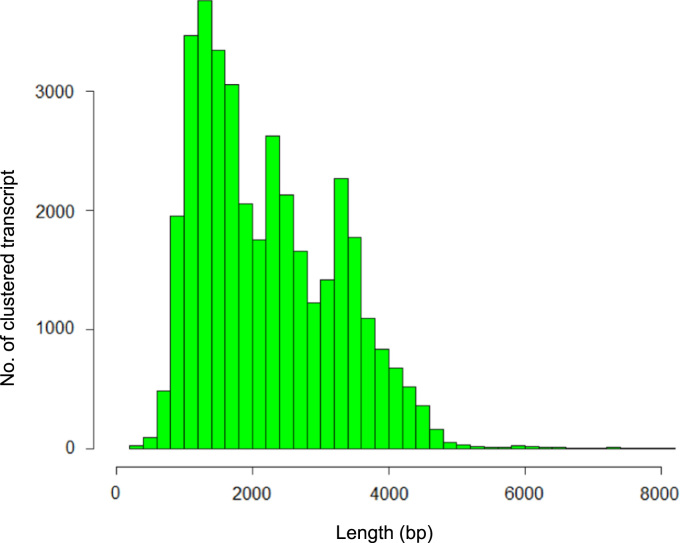
The length distribution of clustered transcripts.

**Fig. 2:**
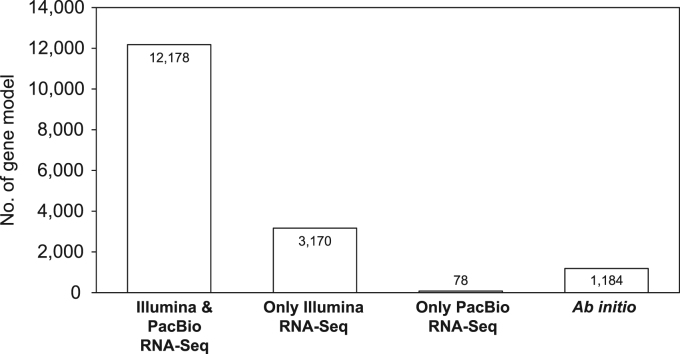
The distribution of gene models supported by PacBio long-read and Illumina short-read RNA-Seq data.

A total of 5,285,247 long-reads producing 12.61 Gb of sequence data were generated from three RNA libraries of the monokaryotic B17 strain of *L. edodes* that were size-selected for lengths of <2 kb, 2–3 kb, and 3–6 kb (Table 1). Those reads were clustered into 36,946 transcripts with a cumulative length of approximately 82.1 Mb and an average length of 2.2 kb (Fig. 1). Based on exon-intron boundary information generated by aligning the PacBio long-read (12.6 Gb) and Illumina short-read (3.36 Gb) [1] RNA-Seq data to the draft genome sequence of *L. edodes* [1], a total of 16,610 protein-coding genes were predicted with error correction (Table 2, Table 3). Of those genes, 1344 were newly identified. The transcriptome data supported 92.9% of the predicted gene models (Fig. 2). Moreover, 7005 gene models (42.2%) were verified to be covered by full-length transcript clusters. Homology-based searches indicated that 76.2% of the predicted genes had homology with known genes. Functional annotations were tentatively assigned for 38.3% of these genes. GFF files and annotations of gene models for *L. edodes* are provided in the Supplementary data (Supplementary material 1 and 2).

**Table 1:** Summary of PacBio long-read transcriptome data in *L. edodes* B17.

	Library size		
	<2 kb	2–3 kb	3–6 kb
No. of subreads^a^	2,027,562	1,404,810	1,852,875
Total length of subreads (Gb)	3.36	3.49	5.76
No. of reads of inserts	196,775	207,733	351,503
No. of full-length reads	91,513	96,258	150,541
No. of non-full-length reads	82,559	99,023	188,716
No. of filtered short reads	22,703	12,452	12,246
Polished consensus isoforms	12,874	11,223	12,849
Average length of isoforms (bp)	1373	2236	3064

a Adapters and artefacts were removed.

**Table 2:** Summary of gene prediction and annotation updated in *L. edodes* B17.

	This study	Shim et al. (2016)
Protein-coding gene (No.)	18,663	13,426
Unique gene models (No.)	16,610	13,028
Genes with isoforms (No.)	2053	398
Supported by RNA-Seq (No.)	15,263	11,781
Annotated (No.)^a^	12,662	10,700
Average gene length (bp)	1288	1612
Total length of gene models (Mb)	24.05	21.64
Exons		
No. of exons	91,386	77,650
No. of average exons per gene	4.89	5.78
Average exon length (bp)	196	204
Introns		
No. of exons	72,723	64,224
No. of average exons per gene	3.89	4.78
Average exon length (bp)	83	90

a Gene models were annotated with homology-based searches.

**Table 3:** Summary of correction of gene models.

	No. of gene models 1
Exactly overlapped	7889
Split into≥two gene models	4742
Fused with≥two gene models	343
Structurally re-predicted	261
Newly found	1344
Predicted in the only previous study	2031

^1^ Gene models in the present study were structurally compared with those reported by Shim et al. [1].

## 2 Experimental design, materials and methods

### 2.1 Materials

The monokaryotic B17 strain of *L. edodes* (KCTC46443) [1] was obtained from the Korean Collection for Type Cultures (KCTC) in the Republic of Korea (http://kctc.kribb.re.kr/).

### 2.2 RNA extraction and PacBio SMRT transcriptome sequencing

Total RNA from the monokaryotic B17 strain of *L. edodes*, which was cultured in potato dextrose broth liquid medium for 10 days at 25 °C, was extracted using an RNA extraction kit (iNtRon Biotech, Seoul, Korea). cDNA was obtained from the RNA and was size-selected into fractions with the following length ranges: 1–2 kb, 2–3 kb, 3–6 kb, and >6 kb. SMRTbell template libraries were created from the obtained cDNAs for sequencing on the PacBio RS II system, as recommended by Pacific Biosciences (Palo Alto, U.S.A.). The templates were sequenced via polymerase binding using the DNA/Polymerase Binding Kit P6 v2 primers.

### 2.3 Long-read transcriptome data clustering, gene prediction and annotation

Long-read transcriptome data clustering was performed using SMRT Analysis software v2.3.0 (https://github.com/PacificBiosciences/SMRT-Analysis) with (i) generation of reads of insert (ROIs), (ii) classification of full-length reads, and (iii) clustering for building consensus sequences.

For gene prediction in the genome of *L. edodes*, AUGUSTUS [2] was used to perform *de novo* prediction with prior gene models trained using GeneMark-ET [3] and exon-intron boundary information predicted by RNA and protein sequence alignments. To generate transcriptome-based evidence, TopHat [4] and GMAP [5] were used for short- and long-read RNA-Seq alignments, respectively. To generate protein-based evidence, homologous protein sequences were collected from the NCBI non-redundant (NR) database, and Exonerate [6] was used for protein sequence alignments to produce protein-based evidence. Predicted genes were searched in the UniProt and NCBI NR databases using BLASTP [7] with a cut-off *E*-value of 1×10^−10^. Protein domains were also searched using InterProScan [8] and then assigned to Gene Ontology (GO) terms.

## Appendix A Supplementary material

Supplementary material 1. Gene models for *L. edodes* in GFF.

.

Supplementary material 2. Annotation of gene models of *L. edodes* in accordance with homology-based searches.
