# Supplementary material for: Long-read transcriptome data for improved gene prediction in Lentinula edodes
Source: Data Brief. 2017 Sep 27;15:454–8. doi: 10.1016/j.dib.2017.09.052 (PMC5961913; doi:10.1016/j.dib.2017.09.052)
Supplement: Supplementary file 1 — Supplementary material [file mmc1.zip › mmc1/Declaration of Interest.pdf]

Declaration of Interest

The authors have no conflicts of interest to declare.
